# Expression of Interleukin-1β and Histological Changes of the Three-Dimensional Oral Mucosal Model in Response to Yttria-Stabilized Nanozirconia

**DOI:** 10.3390/ma16052027

**Published:** 2023-03-01

**Authors:** Naziratul Adirah Nasarudin, Masfueh Razali, Victor Goh, Wen Lin Chai, Andanastuti Muchtar

**Affiliations:** 1 Department of Restorative Dentistry, Faculty of Dentistry, Universiti Kebangsaan Malaysia, Kuala Lumpur 50300, Malaysia; 2 Department of Restorative Dentistry, Faculty of Dentistry, University of Malaya, Kuala Lumpur 50603, Malaysia; 3 Department of Mechanical and Manufacturing Engineering, Faculty of Engineering and Built Environment, Universiti Kebangsaan Malaysia, Bangi 43600, Malaysia

**Keywords:** biocompatibility, interleukin-1, nanozirconia, pro-inflammatory cytokines, three-dimensional oral mucosal model

## Abstract

Over the years, advancement in ceramic-based dental restorative materials has led to the development of monolithic zirconia with increased translucency. The monolithic zirconia fabricated from nano-sized zirconia powders is shown to be superior in physical properties and more translucent for anterior dental restorations. Most in vitro studies on monolithic zirconia have focused mainly on the effect of surface treatment or the wear of the material, while the nanotoxicity of this material is yet to be explored. Hence, this research aimed to assess the biocompatibility of yttria-stabilized nanozirconia (3-YZP) on the three-dimensional oral mucosal models (3D-OMM). The 3D-OMMs were constructed using human gingival fibroblast (HGF) and immortalized human oral keratinocyte cell line (OKF6/TERT-2), co-cultured on an acellular dermal matrix. On day 12, the tissue models were exposed to 3-YZP (test) and inCoris TZI (IC) (reference material). The growth media were collected at 24 and 48 h of exposure to materials and assessed for IL-1β released. The 3D-OMMs were fixed with 10% formalin for the histopathological assessments. The concentration of the IL-1β was not statistically different between the two materials for 24 and 48 h of exposure (*p* = 0.892). Histologically, stratification of epithelial cells was formed without evidence of cytotoxic damage and the epithelial thickness measured was the same for all model tissues. The excellent biocompatibility of nanozirconia, as evidenced by the multiple endpoint analyses of the 3D-OMM, may indicate the potential of its clinical application as a restorative material.

## 1. Introduction

Over the past few decades, zirconia ceramic has been used extensively in dentistry due to its aesthetic property and superior mechanical strength. In clinical applications, 3 mol % yttria-stabilized tetragonal zirconia polycrystals (3Y-TZP) are widely utilized. This is because the yttria-partially stabilized tetragonal zirconia polycrystalline (Y-TZP) showed better mechanical properties and superior resistance to fracture than other conventional dental ceramics [1]. The Y-TZP has a high fracture toughness, from 5 to 10 MPa m^1/2^, and a flexural strength of 900–1400 MPa [2]. The bonding of translucent veneering material to zirconia core materials is usually carried out to cover their opaqueness. However, the most frequently reported complication of zirconia restoration is the chipping or fracture of the veneer ceramic [3]. Henceforward, monolithic zirconia restoration is preferred to overcome such complications.

Nanoparticles can exist between 1 and 100 nm [4,5], and evidence shows that these nanosized particles have a higher surface area-to-volume ratio [6,7]. Hence, this corresponds to the increased particles’ surface energy [8]. Consecutively, nanoparticles have enhanced physical properties compared to their larger-scale counterparts [9]. Due to these exceptionally superior physical properties, nanomaterial applications in dentistry have expanded from caries prevention to restorative dentistry [8,10]. The zirconia dental blocks processed from the nanosized powder have higher translucency than conventional zirconia [11].

So far, toxicological studies on zirconia nanoparticles are limited, and the results are controversial. A study by Laiteerapong et al. [12] reported that zirconia nanoparticles did not have a genotoxic effect on the human gingival fibroblast (HGF). However, evidence from other studies suggested that a reduction in cell viability occurred in a concentration- or time-dependent manner [13,14]. The reduced cell viability was correlated with an increase in the production of reactive oxygen species (ROS), cell death, and significant deoxyribonuclease acid (DNA) damage in the human skin epithelial cells exposed to more than 30 µg/mL yttria-stabilized zirconia nanoparticles for 48 h [15]. Zirconia nanoparticles could also induce a toxic effect on osteoblast cells. The cell proliferation assay demonstrated a decrease in the cell’s viability, with the highest harmful effect at a concentration of 150 μg/mL for 48 h. Additionally, the shape of the osteoblast cells changed, and pyknotic nuclei were observed [16].

Apart from the cytotoxicity testing, it is also essential to investigate the immunogenic potentials of dental materials. Dental materials may provoke local inflammatory reactions of the native gingiva with the induction of pro-inflammatory cytokines. Cytokines are small-sized proteins, normally present in a minimal quantity in body fluids. However, the concentrations can amount to 1000-fold when associated with trauma or inflammation [17]. Cytokines that aggravate inflammation are known as pro-inflammatory cytokines. interleukin-1β (IL-1β) is synthesized by macrophages, monocytes, fibroblast, epithelial and dendritic cells when stimulated by stress or inflammation [18]. Interleukin-1β causes an increased production of cyclooxygenase-2 (COX-2) and phospholipase A2 (PLA2). The increased production, in turn, will activate the prostaglandin E2 (PGE2), leukotrienes, and emigration of neutrophils to the gingiva [19]. As such, increased concentration of interleukin-1β has been implicated in gingival inflammation and bone resorption in periodontal disease [18,20].

The effects of zirconia nanoparticles on the inflammatory response of the human gingival mesenchymal stromal cells (hG-MSCs) were investigated by Nemec et al [21]. They discovered that the addition of zirconia nanoparticles with a size of 100 nm greatly increased the expression of interleukin-6 (IL-6) and interleukin-8 (IL-8) in hG-MSCs. Conversely, no upregulation of IL-6 or IL-1β released by monocytes (THP-1) was observed upon stimulation with zirconia nanoparticles with a diameter of 2–75 µm [22].

With the exponentially increasing clinical use of nanoparticles [23], there is a need to identify the risks concerning human health and the environmental implications of manufactured nanoparticles [16]. Even though studies regarding the toxicity of nanoparticles are rapidly growing, knowledge of the toxic effects of these nanoparticles is limited. Most have agreed that physicochemical characteristics such as shape, surface charge and size play vital roles in nanotoxicity [23,24]. More specifically, grinding activities and wear and tear of the dental restorative materials during function pose a concern that these nanoparticles may leach and cause detrimental effects to the oral cavity and the gingiva.

Nanosized zirconia powders in this study were processed through a combined consolidation method, colloidal slip casting and cold isostatic pressing (CIP) to form zirconia dental blocks [5]. Considering the limited information about cell behaviour with this nanomaterial, utilizing the biological endpoint of 3D-OMMs, this study aimed to assess the biocompatibility of the 3-YZP and to compare it with similar commercially available zirconia.

## 2. Materials and Methods

### 2.1. Preparations of Specimens

Two types of 3 mol% yttria-stabilized tetragonal zirconia were used in this experiment: (1) Sirona inCoris TZI (IC) (Sirona Dental Systems GmbH, Bensheim, Germany), which acted as control; (2) newly developed nanozirconia (3-YZP), prepared from tetragonal zirconia polycrystalline (Y-TZP) nano powder partially stabilized with 3 mol% of Y_2_O_3_ (US Research Nanomaterials Inc., Houston, TX, USA). The primary particle size of the nanopowder was approximated at 20 nm. Briefly, The Y-TZP nanopowder suspension was subjected to the slip-cast method followed by a cold isostatic pressing (CIP) method to produce the green bodies. Subsequently, the green bodies were pre-sintered at 1200 °C to produce the 3-YZP [5]. Both zirconia specimens were trimmed into a disc-shaped form with a dimension of 10 mm diameter and 2 mm thickness. Next, 3-YZP and IC were trimmed to produce a disc-shaped specimen using the high-speed handpiece and straight fissure diamond bur. The zirconia specimens were later polished with sandpaper grit 60, 120, 140, 400, 800, 1200, 1500 and 2000. After final sintering the tested material at 1500 °C, the final dimensions of all samples were approximately 9 mm in diameter and 2 mm in height. Meanwhile, the IC was prepared according to the manufacturer’s instructions.

The surface roughness of the materials was evaluated using the atomic force microscope (AFM) (Park NX-10, Park System, Suwon, South Korea) in contact mode. Six samples were used to determine the surface roughness of the materials. In brief, five random points were chosen in each sample, and these values were averaged.

Before the experiments, all specimens were cleaned with an acetone solution for 20 min in an ultrasonic bath and immersed in the ascending concentrations of ethyl alcohol (10%, 50%, 75% and 100%) each for 5 min. The zirconia specimens were also sterilized on both surfaces under ultraviolet radiation for 20 min (NU-430, LabGard, Class II, Type B2 Biological safety cabinets, NuAire Inc.^®^, Plymouth, MA, USA).

### 2.2. Human Oral Epithelial Cell Line and Human Gingival Fibroblast Cells Culture and Maintenance

The human epithelial cells (OKF6/TERT-2) and human gingival fibroblast cells (HGF) (passage 4–5) used in this study were courtesy of Professor Dr Chai Wen Lin under her research grant supported by the Ministry of Higher Education High Impact Research Grant (UM.C/625/HIR/MOHE/DENT/05). This study received approval from the Research and Ethics Committee, Secretariat of Research and Innovation, Faculty of Medicine, Universiti Kebangsaan Malaysia (UKM PPI/111/8/JEP-2020-618).

The OKF6/TERT-2 cell is an immortalized epithelial cell line processed by the forced expression of the telomerase. This cell line was chosen because it has been proven to resemble the primary oral keratinocytes [25]. The cells were grown in the keratinocyte serum-free media (K-SFM) (Thermo Fisher Scientific Inc., Waltham, MA, USA) with bovine pituitary extract and epithelial growth factor. 

The primary human gingival fibroblast cells were isolated from healthy gingival tissues obtained from the crown lengthening surgery. The primary cell was extracted from the gingival tissue using an explant technique. The Dispase^®^ (Gibco™ Thermo Fisher Scientific Inc., Waltham, MA, USA) was added to the tissue, to separate the connective tissue from the epithelium. The separated tissues were minced into small pieces of 1 mm × 1 mm in size using a scalpel blade in separate Petri dishes and transferred to culture flasks. The growth media in the flasks were changed every 2 days and subcultured once they had attained 70% confluency.

### 2.3. Fabrication of the 3D-OMM

The 3D-OMM was developed based on the modification from the protocols developed by Chai et al. [26]. A mixture of 5 × 10^5^ HGFs and 5 × 10^5^ OKF6/TERT-2 was co-cultured onto the basement membrane side of the rehydrated acellular dermal membrane (Puros^®^ Dermis Allograft Tissue Matrix, Zimmer Biomet, Warsaw, IN, USA). 

Prior to cell seeding, the membrane was cut into a round shape with a 12 mm diameter to fit into a 12 mm ring insert (Corning^®^ Costar^®^ Snapwell™ Insert, Corning Life Sciences, Corning, NY, USA). The membrane was rehydrated in a 5 mL Dulbecco’s phosphate buffer saline (Thermo Fisher Scientific Inc., Waltham, MA, USA) for 30 s and then immersed in a 5 mL DMEM for 15 min. The 3D-OMMs were fabricated inside 12 mm diameter inserts with a 0.4 µm pore size and a 1 × 10^8^ pore density/cm^2^ in a six-well plate. The seeded membrane was submerged in 3 mL of K-SFM and incubated for five days. On the sixth day, the tissues were raised at the air-liquid interface (ALI) to promote epithelial differentiation. The cells were allowed to be stratified further for up to seven days in the incubator, with the media changed every two days. On day 12, the samples (3-YZP and IC) were placed on top of the models. 

### 2.4. Expression of Interleukin-1β

The expression of pro-inflammatory cytokine (IL-1β) from cell culture supernatants of unexposed, 3-YZP-exposed and IC-exposed models were measured using an ELISA kit. Human IL-1β ELISA kit from Abcam PLC, Cambridge, UK (Catalogue Number. ab214025) was used. The cell culture media were collected at 2 time points, after 24 h and 48 h of exposure.

For the assay procedure, all the reagents, controls and samples were prepared based on the manufacturer’s instructions. The optical density was measured using a multi-plate reader (Thermo Scientific™ Multiskan™ Go Microplate Spectrophotometer, Thermo Fisher Scientific, MA, USA) at 450 nm and correction at 570 nm. The concentration of IL-1β was expressed as pg/mL and compared with a standard curve provided by the manufacturer.

### 2.5. Histology Preparation

Histological analyses of the 3D-OMMs were performed after 72 h of contact. The models were fixed in a 10% formalin solution for 24 h, processed for histological sections via dehydration in a graded series of ethanol concentrations (70–100%) and embedded in a paraffin block. Vertical sections of 5 µm thickness were cut and stained with hematoxylin and eosin staining (H&E staining).

The resultant histology sections were evaluated for evidence of cytotoxic epithelial damage using the inverted microscope (IX51 Olympus, Olympus Corporation, Shinjuku, Japan) at 20× and 40× magnification. The damage was manifested as the loss of the normal cell’s morphology, such as the separation of the epithelium from a scaffold and the presence of the pyknotic nuclei. Two examiners were assigned to examine the histological appearance of the models.

The thickness of the epithelial layers of all models was measured using the imaging software (Cell^B^, Olympus Soft Imaging Solutions GmbH, Imaging Software, Muenster, Germany) at 20× magnification. A linear scale was placed perpendicular to the histology image. The distance was taken from the basement membrane’s basal surface to the epithelium’s uppermost keratinized layer. Measurements were taken at five random points per section, and these values were averaged.

### 2.6. Statistical Analyses

All experiments were conducted in triplicate. The thickness of epithelial layers and the concentration of IL-1β were expressed as mean data and standard deviations. The data were analyzed with IBM SPSS version 25. The Shapiro–Wilk normality test was used to determine normally distributed data with *p*-values > 0.05. Levene’s test and Box’s M test were used to test the assumption of homogeneity of variance and covariances. A two-way mixed ANOVA and post hoc test were used to analyse the mean concentration of IL-1 β expression, the difference between and within the models. While a one-way Welch ANOVA and post hoc test were used to assess the differences in models’ epithelial thickness. The hypothesis of no difference was accepted when *p*-values > 0.05.

## 3. Results

### 3.1. Surface Roughness of the Materials

**Figure 1:**
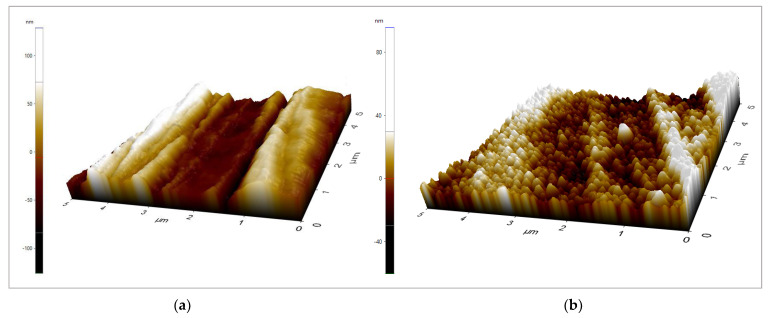
The surface topography of (**a**) 3−YZP and (**b**) IC surfaces., areas of 5 µm × 5 µm were captured. The z scale bar (**a**) −100–100 nm; (**b**) −40–80 nm.

The individual components of each sample and the surface roughness values of two zirconia discs are tabulated in Table 1. All materials surfaces were categorized within the range of smooth surface values (Ra 0.0–0.5 µm). There was no statistically significant difference (*p* > 0.05) between the two types of zirconia. Figure 1 shows the topographic surface profile of each material used in this study, as scanned by AFM.

### 3.2. Expression of the Interleukin-1β following Exposure to 3-YZP

**Figure 2:**
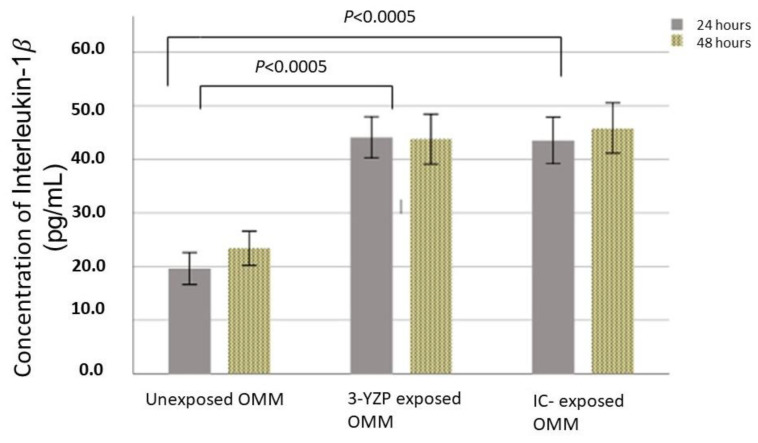
IL-1β released after exposure of the 3-D oral mucosal model to 3-YZP and IC, after 24 and 48 h. Error bars represent standard deviation.

Figure 2 shows the concentration of IL-1β expressed when in contact with 3-YZP and IC. For 24 h of contact, the unexposed model released a statistically significantly lowered amount of IL-1β (19.59 ± 3.88 pg/mL) compared to both 3-YZP-exposed (44.09 ± 3.65 pg/mL) and IC-exposed (43.52 ± 4.14 pg/mL) oral mucosal models. The difference was also significant for 48 h of contact compared to the blank model. The concentration of IL-1β measured after 48 h on contact was 23.38 ± 4.17 pg/mL, 43.74 ± 4.45 pg/mL and 45.85 ± 4.47 pg/mL for the unexposed model, 3-YZP-exposed and IC-exposed oral mucosal model, respectively. However, the IL-Iβ expression between 3-YZP and IC was not statistically significant (0.77, 95% CI (−3.5 to 5.07), *p* = 0.892). It is interesting to note that from this chart (Figure 2), the prolonged contact with 3-YZP did not increase the amount of IL-1β released.

### 3.3. Histological Sections of 3D-OMMs

**Figure 3:**
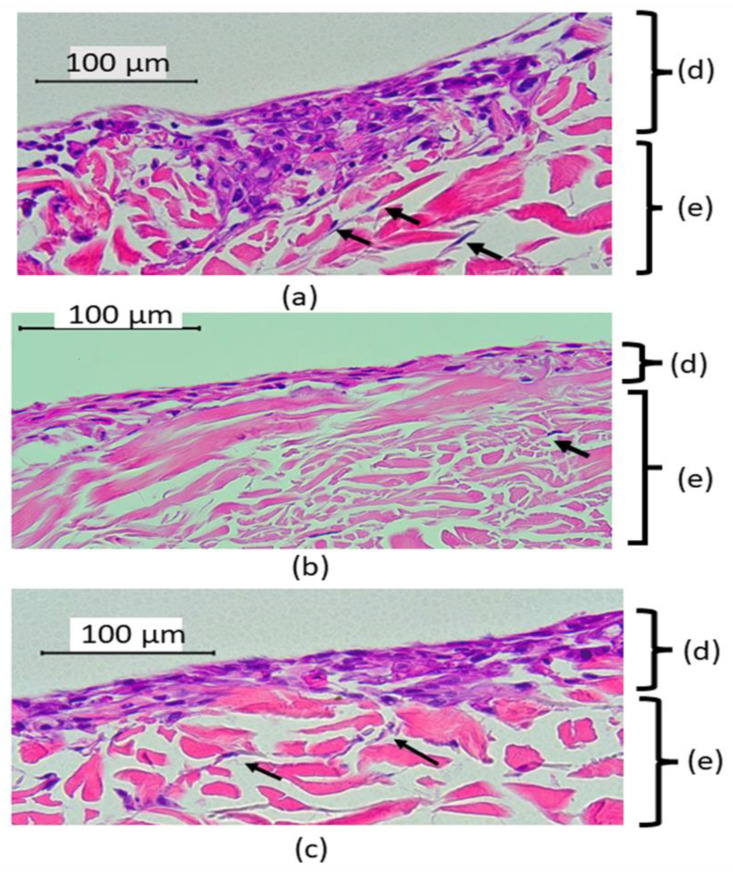
Histological appearance of 3D-OMMs, unexposed (**a**), after 72 h exposed to 3-YZP (**b**) IC (**c**). Black arrows indicate that the HGFs migrated into connective tissue and were occasionally present inside the stroma (**e**). The epithelial layers (**d**) were inconsistent. (Original magnification, 40×).

This study fabricated the full-thickness model that contained both types of cells. The acellular dermal membrane used in this study allowed the migration of the fibroblasts (indicated by black arrows in Figure 3) within the membrane and the fibroblasts played an important role in modulating epithelial cell differentiation, thus mimicking the intra-oral situation in which the cells interact with materials. The resultant histology sections were evaluated for evidence of cytotoxic epithelial damage using a light microscope at 40× magnification. Histologically, the epithelium continuity was preserved in all models. The absence of pyknotic nuclei was observed, and the integrity of the connective tissue layer was intact. The epithelial layers of 3D-OMMs were inconsistent, while the HGFs were not homogenized after seeding into the acellular dermal scaffolds. All hematoxylin and eosin staining results of 3D-OMM are shown in Figure 3.

**Figure 4:**
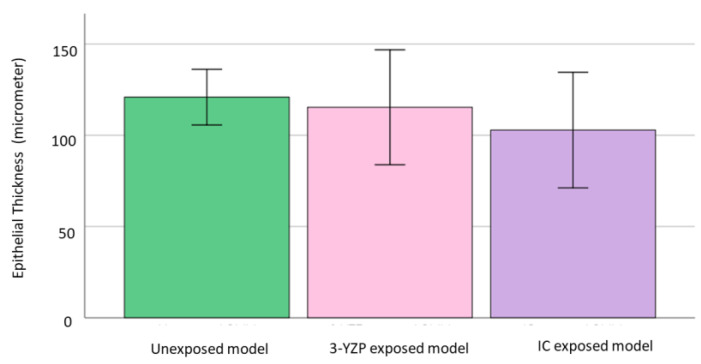
Graph reported on the epithelial thickness of unexposed model, 3-YZP and IC-exposed model. Error bars indicate standard deviation (SD).

The epithelial thickness was highest in the unexposed group (120 ± 19.8), followed by 3-YZP-exposed (115.34 ± 41) and IC-exposed (102.84 ± 41.2). The differences between these groups were not statistically significant, Welch’s F (2,14.0) =0.68, *p* = 0.523. Figure 4 shows the epithelial thickness of the unexposed model, 3-YZP, and IC-exposed models.

## 4. Discussion

Surface roughness typically plays an essential role in cell attachment and proliferation [27,28]. In our study, the surface roughness was standardized via the polishing method to reduce a possible confounding factor in cell attachment. Additionally, surface roughness promoted biofilm formation. Others have shown that the surface of an abutment should have a moderate smoothness to hamper plaque formation and permit cell adhesion. With that being said, an optimal surface roughness threshold of Ra 200 nm (0.2 µm) has been proposed [29,30]. In this study, the surface roughness of all materials was within the optimum surface roughness. Additionally, the Ra value obtained from manual polishing in this present study was consistent with the value obtained from another author [31]. Although 3-YZP exhibited a slightly higher Ra value than IC, the Ra values were not more than the threshold proposed.

The 3-YZP and IC had acceptable tetragonal phase stability. The X-ray diffraction (XRD) test revealed a negligible amount of monoclinic zirconia after sintering [5,11,32]. An increased amount of monoclinic phase zirconia was found when the IC was subjected to accelerated hydrothermal ageing at 124 °C [33]. However, the monoclinic zirconia content after ageing was not exceeded the proposed engineering guidelines for zirconia restoration [34]. The presence of a high level of monoclinic phase is associated with the problem of low-temperature degradation (LTD). The LTD results in surface micro-cracking, increased surface roughening, grains pull-out and degrades the mechanical properties of the dental crown [35]. In a different study [36], where tested zirconia undergone (CIP) produced a tetragonal phase content of 100% and better mechanical properties, higher density and more homogeneous microstructures than zirconia that were not subjected to CIP. Additionally, the pre-sintered block at 1200 °C exhibited the lowest shrinkage (9.0%) among all the samples after the blocks were further sintered to a final temperature of 1500 °C with heating and cooling rates of 3 °C/min and a holding time of 2 h [37].

Recently, studies have incorporated the pro-inflammatory mediator’s assessment for cytotoxic changes in cells that directly interact with dental materials [38,39,40]. The IL-1β was selected in this study as it is a potent pro-inflammatory mediator and is easy to measure. Moreover, the nanomaterials have the potential to trigger the multiprotein complexes and inflammasome, which will result in the secretion of IL-1β [41]. Our results demonstrated that the untreated oral mucosal model released some amount of IL-1β. This was consistent with the findings in previous studies [42,43,44]. Furthermore, our analysis found a significantly increased amount of IL-1β released when 3D-OMMs were exposed to both 3-YZP and IC. Nevertheless, the difference between both ceramics was insignificant.

The in vitro studies of pro-inflammatory mediators among zirconia restorations, especially utilizing the 3D-OMMs, are limited. In line with our result, a study by Özen et al. [45], who investigated the effects of dental alloys and ceramics on the oral mucosal model, revealed that In-Ceram had no significant influence on IL-1β at 24 h of exposure. However, after 48 h, the amount of IL-1β was about two-fold higher than those released from control cultures. This indicated that the dental ceramic may have caused an inflammatory reaction to the tissue model, but no toxic effect was evident as no significant reduction in cell viability was reported. Another postulation for the upregulation of the IL-1β expression was that the nanomaterials irritated the epithelium of the 3D-OMMs and caused the inflammatory response [46]. The epithelial cells could secrete pro-inflammatory mediators such as IL-1β and IL-6, in response to inflammation or tissue injury. Prior research concluded that most IL-1β has been found in the gingival epithelial [18].

Presently, there is no direct evidence that an increased IL-1β released after exposure to ceramic indicates inflammatory reactions in vivo. The ceramics’ immunogenic potential was mainly reported in human clinical studies by estimating the inflammatory mediators in the gingival crevicular fluid (GCF) [47,48,49]. Therefore, GCF was chosen as an indicator for periodontitis and health. Ariaans et al. [50], who aimed to quantify the inflammatory reaction between lithium disilicate and zirconia, discovered that the gingival reaction between both materials was comparable to the unrestored tooth of periodontally healthy patients. The findings of our study were in agreement with Saravanakumar et al. [51], who found that, after three months, the zirconia crown expressed the lowest amount of IL-1β in the GCF, among other restorations. However, various factors could have influenced human clinical studies; so, comparing their findings with our research is challenging.

In this current research, we assessed the toxicity of the 3-YZP by placing it directly onto the uppermost surface of the oral mucosal models as an experiment by Moharamzadeh et al. [42]. Histological evaluation is currently the gold standard for toxicity assessment of imaging tissue-engineered models [52]. Highly toxic materials can cause discontinuation of the epithelial layer and separation of the epithelium from the basement membrane, some may show marked architectural atrophy and most of the upper cell layers were disintegrated but the basal cells remained intact with connective tissue components [53]. Histological analysis of all models in this study revealed that the epithelial layers were continuous, and the integrity of the connective tissue was preserved. However, only some fibroblasts were seen inside the matrices. Although acellular dermal matrices (ADM) are widely accepted as a scaffold, it suffers from some limitations due to their low porosity, which causes the poor distribution of the fibroblasts inside the connective tissue [54,55,56]. It is generally accepted that fibroblasts promote epithelial differentiation. However, the dermal matrices are unique as the basement membrane on the epidermal side of the matrices promotes oral keratinocytes adherence better than the collagen matrices.

Our analysis of epithelial thickness revealed that the epithelial thickness was the highest for the untreated models, followed by the 3-YZP-exposed and IC-exposed models. However, the one-way Welch ANOVA failed to demonstrate the statistically significant difference between these groups. Furthermore, the reduction in the epithelial thickness of the model correlated with the increased rate of cell apoptosis [44]. Hence, the thickness of the model can be extrapolated for the quantitative analysis of the histological appearance to determine the material’s toxicity. Our result was suggestive that 3-YZP had no toxic effect on the soft tissue, as the model’s epithelial thickness was preserved.

With the increasing clinical use of nanoparticles, there is a need to identify risks concerning human health [57]. Currently, the conventional in vitro test employed for cellular biology and toxicology is used to assess nanotoxicity. There are many ways of biological effects can be assessed including cellular attachment and migration, proliferation rates and determination of enzyme activity. However, due to the unique physicochemical properties of nanomaterial, interferences of nanomaterial with conventional toxicity assays such as monolayer cell cultures may have been reported and led to questionable results for nanotoxicity. Notwithstanding, the use of 3D-OMM resembling the physiological environment of the oral cavity and being used for biocompatibility testing could serve as transition testing between monolayers, to preclinical testing. The release of inflammatory cytokines such as interleukin-8 (IL-8) and interleukin-6 (IL-6) can significantly modify the cell viability and enzymic activity of the tissue model [25]. The oxidative imbalance induced by nanosize dental materials zirconia may stimulate apoptosis, immunological, increased expression of oxidative stress [58] and carcinogenic effects in the oral epithelium of 3D-OMM [59,60]. In the oral cavity, the nanozirconia dental crown will be placed in close contact with the gingiva and oral mucosa for prolonged periods, the physiological environment may induce local toxic effects. The nanozirconia dental crown may lead to chemically or pathologically initiated inflammatory diseases such as gingivitis and periodontal disease. Therefore, the re-engineered organotypic model of the oral mucosa will provide indispensable fundamental knowledge in understanding the physiological functions of tissues and the biological response of dental materials. The use of 3D-oral mucosal models could offer more predictable, repeatable and reliable multiple endpoint analyses of nanomaterials in dentistry, which mimic the in vivo morphology and cell behaviour. Moreover, they constitute a cheaper and ethically acceptable alternative to animal testing; therefore, a genotoxicity assessment should be carried out in future, utilizing tissue-engineered equivalents.

## 5. Strengths and Limitations of the Study

The in vitro biocompatibility assessment is crucial for the clinical validation of dental materials, which allows the evaluation of many samples simultaneously. The evidence regarding the biocompatibility of zirconia on monolayer cells has been discussed extensively in the literature. The use of 3D-OMM could serve as transition testing between monolayers, to preclinical testing, where mucotoxicity and genotoxicity may resemble those in vivo due to multiple cellular interactions that could be observed. Nevertheless, the evidence of zirconia toxicity on oral mucosal equivalent is limited. Using the 3D-OMM would allow the investigation of the inflammatory mediators and the histological evaluation of the changes in soft tissue in response to the toxicity of dental nanomaterials. Additionally, the tissue culture assays will provide a controllable and repeatable method of assessment. Hence, the findings reported from the analysis of the oral mucosal model in this study are more clinically applicable than the monolayer cell culture.

However, this study also had some limitations. The histological analysis of the models was assessed by more than one investigator who was not blinded to the nature of the treatment the tissue had received. This could have potentially caused bias. Furthermore, only one pro-inflammatory cytokine was reported in this current research. The potential use of other interleukins, such as IL-6 [61], IL-8 and tumour necrosis factor-α [62] should be explored in the biocompatibility testing of the newly developed material. The IL-6, the pro-inflammatory cytokine, is also essential for mitogenic and proliferative effects on keratinocytes during wound healing and tissue remodelling [61]. 

In addition to that, the nanoparticle’s size, shape, surface area, agglomeration state, chemical composition and surface charge may influence their toxicity and biological responses. Among the various microscopy method for physicochemical characterization, the scanning electron microscope, transmission electron microscope (TEM) and atomic force microscope are frequently used. With this regard, TEM can be used to evaluate the permeation effects of the cell in contact with nanomaterials. Meanwhile, single-particle ICP-MS can be used for information on the size and size distribution of nanoparticles or the number of leachable elements from the nanozirconia. Even though both the zirconia were stabled following sintering, the oxide bonds of the ceramic could be damaged by the ageing or LTD of the zirconia. A study had shown that some yttrium ions were leaching out from the commercial zirconia in the corrosion test [63]. Nevertheless, the amount of leached yttrium ions as measured from the ICP-MS was relatively low than the level that could potentially be toxic to humans. 

## 6. Conclusions

Within the limitations of this study, the excellent biocompatibility of the 3-YZP, as shown from the biological endpoint analyses of the 3D-OMM, may indicate the potential of its clinical application as a dental restorative material. It was found that 3-YZP supported oral fibroblasts and keratinocytes’ cellular attachment and proliferation. There was no cytotoxic damage to the human oral mucosal equivalent, as demonstrated histologically with the preservation of the epithelium layer. However, the 3-YZP had the potential to modulate the secretion of IL-1β, similar to the currently used zirconia (IC). 

## Figures and Tables

**Table 1:** The composition and surface roughness of each material.

Materials	Components and Composition by Weight %	Mean Surface Roughness ± (SD) (Ra) (nm)	*p*-Value
Nanozirconia (3-YZP)	ZrO_2_/Y_2_O_3_/HfO_2_ 95%/<5%/<1	93.8 ± 52.5	0.794 *
inCoris TZI (IC)	ZrO_2_/Al_2_O_3_/Y_2_O_3_	85.9 ± 49.5	

* Both Ra values of 3-YZP and IC were not statistically significant. SD = standard deviation; ZrO_2_ = zirconium oxide; Y_2_O_3_ = yttrium oxide; HfO_2_ = hafnium dioxide; Al_2_O_3_ = aluminum oxide.

## Data Availability

The data presented in this study are available on request from the corresponding author.
